# Physiological Effects of Deoxynivalenol from Naturally Contaminated Corn on Cerebral Tryptophan Metabolism, Behavioral Response, Gastrointestinal Immune Status and Health in Pigs Following a Pair-Feeding Model

**DOI:** 10.3390/toxins13060393

**Published:** 2021-05-30

**Authors:** Yan-Bin Shen, Alexandra C. Weaver, Sung Woo Kim

**Affiliations:** 1 Department of Animal Science, North Carolina State University, Raleigh, NC 27695, USA; Yanbin.Shen@apcproteins.com (Y.-B.S.); aweaver@alltech.com (A.C.W.); 2 APC, Ankeny, IA 50023, USA; 3 Alltech, Inc., Nicholasville, KY 50023, USA

**Keywords:** behavior, deoxynivalenol, gut morphology, nursery pigs, serotonin, tryptophan

## Abstract

This study investigated the impact of deoxynivalenol (DON) from naturally contaminated feed on pig growth, immune status, organ health, brain serotonin (5-Hydroxytryptamine, 5-HT) and behavior. Sixteen individually housed pigs (25.57 ± 0.98 kg, age 9 weeks) were randomly allotted to two dietary treatments: without DON (CON) or with 3.8 mg/kg of DON (MT). Pigs were pair-fed to eliminate differences in feed intake (equal tryptophan (Trp) intake). Pigs fed CON received a daily ration based on the *ad libitum* feed consumption of their MT pair-mate. Performance was determined over 21 days and blood collected for immunological and oxidative stress parameters. Behavior was recorded for 12 h on days 0, 7, 14 and 21. After 21 days, pigs were euthanized to collect tissues for immune parameters, gut morphology and brain serotonin levels. Overall, pigs fed MT had greater weight gain compared with CON. Immunological and oxidative stress parameters were unaffected, but pigs fed MT had reduced villus height, crypt depth and villus-to-crypt ratio in the jejunum. Pigs consuming MT had reduced concentration of 5-HT and increased 5-HT turnover in the hypothalamus. Mycotoxin-fed pigs spent more time lying and sitting, and less time standing and drinking. In conclusion, consumption of DON impacted gastrointestinal tract structure, altered behavior and changed Trp metabolism through increasing 5-HT turnover in hypothalamus.

## 1. Introduction

Deoxynivalenol (DON), a member of the type B trichothecene mycotoxins, is one of the most prevalent mycotoxins worldwide [1,2,3]. Due to its high global occurrence, DON is regarded as an important risk factor for animal health [4]. It is well known that DON causes a variety of damages such as altered protein synthesis, gastrointestinal injury and altered immunity [5,6]. However, the most widely recognized characteristic of DON consumption is its detrimental effect on feeding behavior. High levels of DON can trigger vomiting, whereas lower levels induce anorexia [5]. Reducing feed intake may indirectly result in limited nutrient availability, causing growth reduction, digestive disorders and immune challenges [7]. 

Whereas the exact mechanism behind the detrimental effect of DON on feed intake is not fully understood, several studies have suggested that altered metabolism of the dietary essential amino acid tryptophan (Trp) and the serotoninergic system in the brain may be the cause [8,9,10,11]. The cerebral neurotransmitter serotonin (5-hydroxytryptoamine, 5-HT), synthesized from Trp, plays a major role in regulating appetite [12]. It has been proposed that DON increases levels of peripheral and brain Trp, as well as 5-HT, and consequently reduces feed intake [13,14]. However, simply increasing 5-HT levels in the whole brain cannot explain the vomiting and anorexia effects of DON, as enhancing 5-HT in the brain by adding high levels of Trp is repeatedly shown to have no effect on feed intake [15,16,17,18]. Therefore, it is hypothesized that exposure of pigs to naturally contaminated DON can alter Trp metabolism, with varying actions in different brain regions leading to feed reduction and behavioral changes. It is also hypothesized that the associated physiological changes caused by DON will damage intestinal morphology, alter immunity and impact pig growth. Thus, the objective of this study was to investigate the dietary exposure of DON on 5-HT concentrations in different regions of the brain, as well as to determine the health-related changes in pigs following DON consumption. 

## 2. Results

### 2.1. Growth Performance 

The initial body weight (BW) of pigs did not differ between treatments. During the first 7 d, pigs fed with the MT diet had greater (*p < 0.05*) body weight gain and gain:feed compared with pigs fed with the CON diet (Table 1). During the entire 21-day period, pigs fed with the MT diet had greater (*p < 0.05*) body weight gain compared with pigs fed with the CON diet. In agreement with the pair-feeding procedure, average daily feed intake (ADFI) of pigs was not different between treatments.

### 2.2. Physical Activity and Salivary Cortisol 

**Figure 1:**
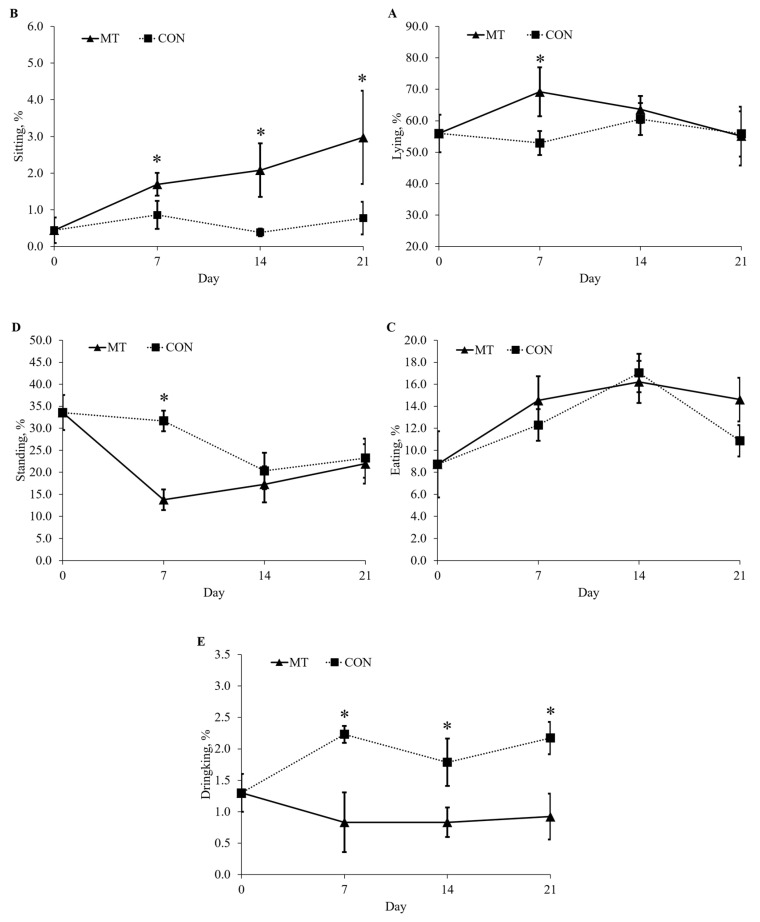
Behavioral activities of lying (**A**), sitting (**B**), eating (**C**), standing (**D**) and drinking (**E**) 9-week-old nursery pigs fed either a diet without deoxynivalenol (CON) or a diet with 3.8 mg/kg of naturally contaminated deoxynivalenol (MT). An instantaneous scan-sampling method with 1 min intervals was used to determine percentage of time spent on various behaviors for every other hour from 8:00 a.m. to 8:00 p.m. on day 0, 7, 14 and 21 of the experiment. Each data point represents the mean ± SE of 8 barrows. * *p* < 0.05.

**Figure 2:**
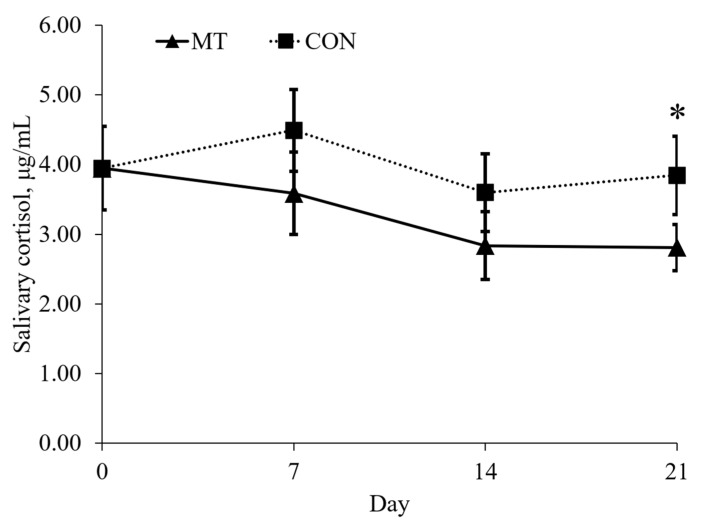
Concentrations of salivary cortisol of 9-week-old nursery pigs fed either a diet without deoxynivalenol (CON) or a diet with 3.8 mg/kg of naturally contaminated deoxynivalenol (MT). Salivary cortisol was analyzed on day 0, 7, 14, and 21. Each data point represents the mean ± SE of 8 barrows. * *p* < 0.05.

Pigs fed with the MT diet spend more time lying (*p* < 0.05) and less time standing (*p* < 0.05) compared with pigs fed with CON on day 7 (Figure 1). On day 7, 14, and 21, pigs fed with the MT diet spent more time sitting (*p* < 0.05) and less time drinking (*p* < 0.05) compared with pigs fed with CON. On day 21, pigs fed with the MT diet had lower (*p* < 0.05) concentration of salivary cortisol compared with pigs fed with CON (Figure 2).

### 2.3. Immunological and Oxidative Stress Analysis

Plasma immunological parameters for IgA, IgG, IgM and TNFα were not different between treatments for day 7 or 21 (Table 2). The concentration of plasma malondialdehyde (MDA), as a measure of lipid peroxidation, also did not differ between treatments for either time measurement. Analysis of the stomach, duodenum, jejunum and ileum tissues indicated that IgA, IgG and IgM did not differ between treatments (Table 3). Only jejunum tissue had increased (*p* < 0.05) TNF-α levels.

### 2.4. Gut Morphology

The depth of the gastric pits in the stomach were decreased (*P < 0.05*) in pigs fed with the MT diet. Analysis of intestinal morphology indicated that villus height and crypt depth was reduced (*p* < 0.05) in the jejunum of pigs fed with the MT diet (Table 4). Villus height in the ileum tended to be decreased (*p* = 0.07) in pigs fed with the MT diet. These changes in villus and crypt morphology lead to a reduced ratio (*p* < 0.01) of villus height to crypt depth in both the jejunum and ileum in pigs fed with the MT diet. The morphology of the duodenum was unaffected by dietary treatments.

### 2.5. Hypothalamic Serotonin and 5-Hydroxyindoleacetic Acid

**Figure 3:**
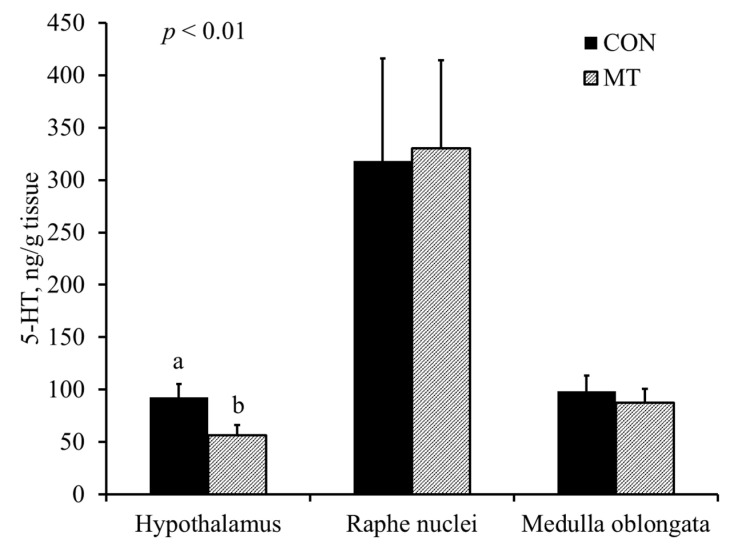
Serotonin (5-HT) levels in different regions of the brain of 9-week-old nursery pigs fed either a diet without deoxynivalenol (CON) or a diet with 3.8 mg/kg of naturally contaminated deoxynivalenol (MT). Each bar represents the mean ± SE of 8 barrows. ^a,b^ Values with different letters are different at *p* < 0.01.

**Figure 4:**
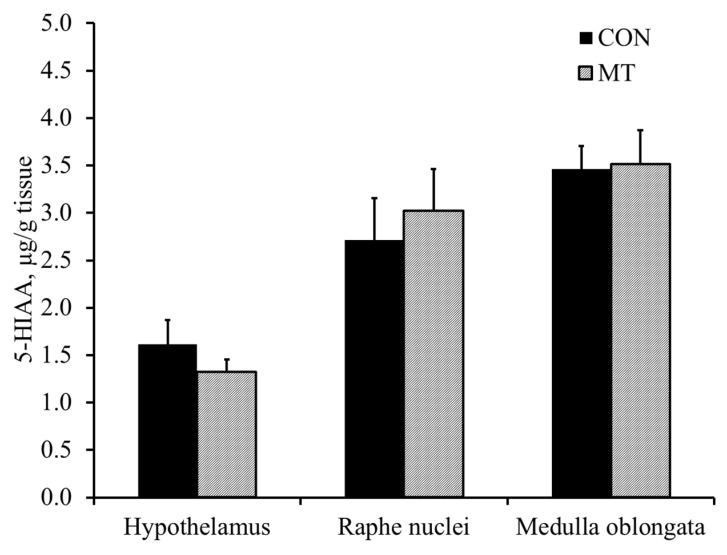
5-hydroxyindoacetic acid (5-HIAA; metabolites of serotonin) levels in different regions of the brain of 9-week-old nursery pigs fed either a diet without deoxynivalenol (CON) or a diet with 3.8 mg/kg of naturally contaminated deoxynivalenol (MT). Each bar represents the mean ± SE of 8 barrows.

**Figure 5:**
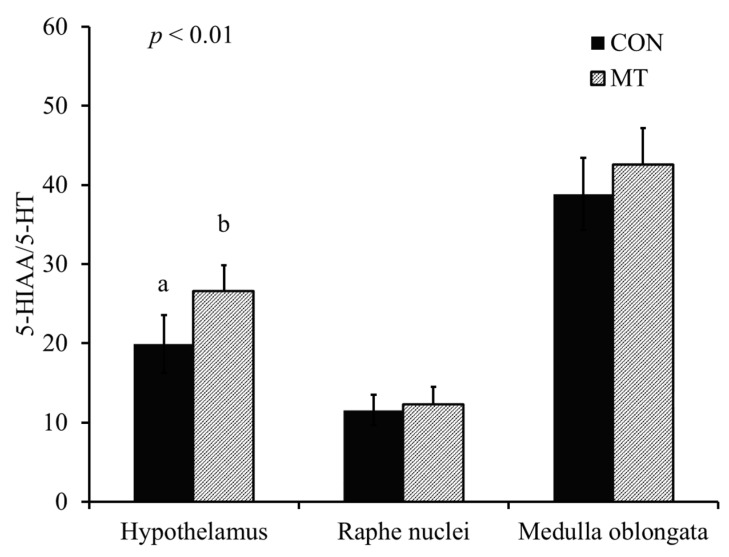
Serotonin turnover index (5-hydroxyindoacetic acid (5-HIAA)/serotonin (5-HT)) in different regions of the brain of 9-week-old nursery pigs fed either a diet without deoxynivalenol (CON) or a diet with 3.8 mg/kg of naturally contaminated deoxynivalenol (MT). Each bar represents the mean ± SE of 8 barrows. ^a,b^ Values with different letters are different at *p* < 0.05.

Pigs fed with the MT diet had reduced (*p* < 0.01) concentration of 5-HT in the hypothalamus compared with pigs fed with the CON diet (Figure 3). There was no difference in the concentration of 5-hydroxyindoacetic acid (5-HIAA) between different treatments (Figure 4). Pigs fed with the MT diet had an increased (*p* < 0.01) 5-HT turnover index (5-HIAA/5-HT) in the hypothalamus compared with pigs fed with the CON diet (Figure 5).

## 3. Discussion

Serotonin, synthesized from Trp, is closely involved in the central regulation of feeding behavior [12]. The rate-limiting step of 5-HT biosynthesis is the hydroxylation step of the methoxyindole pathway, which is not saturated by L-Trp in vivo as the Km for Trp is about 50 µg/g tissue and the prevailing concentration of Trp in brain is 5 µg/g tissue [19]. Therefore, the ability of Trp as a substrate is the rate-limiting factor of 5-HT biosynthesis in the brain. Several studies have linked the anorexic activity of DON to central nervous system serotoninergic activity [8,9,10]. Studies have reported that oral administration of DON increased levels of peripheral and brain Trp and, consequently, 5-HT in the brain of rats [13,14,20]. In these trials, it was concluded that the increased level of 5-HT in the brain was the reason for DON-induced feed refusal. 

However, simply increasing 5-HT levels in the brain does not necessarily lead to feed refusal. Shen et al. [18,21] reported that dietary supplementation of L-Trp increased hypothalamic 5-HT concentration but did not affect feed intake in pigs. Similar results have been confirmed by other studies [15,16,17]. Moreover, Prelusky [22] reported that DON had no effect on concentrations of plasma 5-HT and Trp in pigs. One study with pigs has even shown that intravenous injection of DON reduced 5-HT in the hypothalamus in the long term [8]. Thus, the neuropharmacology of DON may be more complicated than originally assumed. In the current trial, pigs fed CON were pair-fed to pigs in MT in order to keep the Trp intake similar between treatments. Pigs fed a diet with DON had reduced hypothalamic 5-HT and an increased 5-HT turnover rate compared with pigs fed a diet without DON, clearly indicating that DON caused catabolism of 5-HT and interfered with L-Trp metabolism. 

The serotonergic neurons are the major constituents of the raphe nuclei, and most of the neurons of the raphe nuclei are the principal source of 5-HT release in the brain [23]. The 5-HT projection pathway of serotonergic neuron from raphe nuclei to various parts of the brain results in different physiological and behavior reactions [24]. The 5-HT projection pathways to the hypothalamus mediates anorexia and feeding behavior, whereas 5-HT projections to medulla oblongata may mediate vomiting [24]. One of the major findings of our current study is that dietary exposure to 3.8 mg/kg of DON reduced the concentration of 5-HT and increased the 5-HT turnover index (5-HIAA/5-HT) in the hypothalamus, whereas concentrations of 5-HT, 5-HIAA and 5-HT turnover index in the raphe nuclei and medulla were not affected. To the best of our knowledge, this study is the first to date that report the effect of feeding naturally DON-contaminated grains on 5-HT activities in different regions of the brain in pigs. This result is supported by Prelusky [9], who observed a significant and prolonged increase in 5-HIAA in the cerebral spinal fluid of pigs following intragastric administration of purified DON, indicating enhanced 5-HT turnover. Moreover, Swamy et al. [11] reported that feeding a blend of *Fusarium* mycotoxins (5.5 mg/kg DON, 0.5 mg/kg 15-acetyl-deoxynivalenol, 26.8 mg/kg fusaric acid and 0.4 mg/kg zearalenone) also increased the 5-HT turnover index. Studies have also shown that increased projection pathways of 5-HT into the hypothalamus results in feed refusal [25]. Thus, it is proposed that the increased turnover index of 5-HT in the hypothalamus is one of the mechanisms of DON-induced feed refusal. 

Serotonin also plays a major role in social interactions and behavioral responses in humans and domestic animals [26,27,28]. Altered hypothalamic 5-HT of pigs by chronic DON exposure may change the behavior of pigs. Studies have shown that reduced 5-HT production could result in depression which, in pigs, could be considered as passive and quiescent behavior [29]. In our current trial, pigs fed a diet with DON spend more time lying as well as sitting, and less time drinking and standing, showing this passive and quiescent behavior after DON exposure. Those results indicate that increased catabolism of 5-HT altered the behavior of pigs by reducing activity. 

Reduced feed intake, vomiting and decreased growth rates are well known effects of DON intake by pigs [30,31]. Research has shown that feed intake can be reduced by over 35% at DON levels of 4.8 to 5.6 mg/kg in a diet [11,32,33]. In these trials, average daily gain (ADG) was also reduced by 33%. Interestingly, in our current experiment, pigs fed MT had greater weight gain compared with pigs fed the CON diet. Since pigs were pair-fed, increased weight gain may have been due to increased feed efficiency. These results are consistent with a study by Rotter et al. [34], which reported that a 3.0 mg/kg dietary inclusion of DON increased body weight gain and feed efficiency of pigs when pigs were pair-fed. Rotter et al. [34] also reported that feed efficiency was linearly increased with an increased dietary inclusion of DON from 0.0 to 3.0 mg/kg in a diet, whereas body weight gain and feed intake decreased linearly. The mechanism underlying the increased feed efficiency of DON exposure is largely unknown, but presumably relates to 5-HT and behavior activities of pigs. As pigs decrease their physical activity, their maintenance requirements may also be reduced, thereby improving efficiency. Additionally, pigs fed CON were observed to consume the entirety of their daily ration within 30 min after feeding in the morning, whereas pigs fed MT ate small amounts throughout the day. This also may have altered the feed efficiency and response in growth performance.

Beyond the impacts on brain 5-HT turnover and behavior, DON is also shown to impact protein synthesis, intestinal health and immune functions [2,6,35]. However, the effects on these systems by DON are not always clear. Weaver et al. [36] did not observe significant changes in serum immunoglobulin concentrations when pigs consumed a combination of 150 µg/kg aflatoxin and 1.1 mg/kg DON, although there was a tendency for increased immunoglobulins after 42 days. Swamy et al. [37] also did not observe changes in serum immunoglobulin concentrations using a pair-feeding model where pigs consumed DON and zearalenone. In contrast, Goyarts et al. [7] indicated that serum IgA significantly increased when pigs consumed DON ad libitum but not when pigs were restrictively fed equal amounts of control and contaminated diets. In our current study, DON contamination did not impact immunoglobulin levels, indicating that there was no change in the antibody response of the immune system. Despite there being no changes in the innate immune response, as measured by the immunoglobluin production, there was an increase in the production of the proinflammatory cytokine, TNF-α, in the jejunum tissue of pigs fed MT. This increase in TNF-α may indicate an acute-phase inflammatory reaction caused by the toxic effects of DON on the cellular lining of the jejunum. Previous research has also indicated that TNF-α concentrations can increase following the intake of 180 µg/kg of aflatoxin and 0.9 mg/kg of DON by pigs [38]. In contrast, no change in TNF-α was observed when pigs consumed a diet containing 230 µg/kg of aflatoxin with 8.99 mg/kg of fumonisin [39]. Thus, the observed changes in TNF-α in our current study, and in Chaytor et al. [38], may likely be a result specifically of DON intake.

Although immune status was not greatly affected by DON, gut morphology data did indicate that DON caused damages to the gastrointestinal tract. In our study, there was some change beginning in stomach lining, where there was a reduction in the depth of the gastric pits; however, it is the villi structure that appeared to experience the most damage due to the DON challenges, with villus height reduced in both the jejunum and ileum. Crypt depth was also increased in the jejunum, and in both the jejunum and ileum the villus-to-crypt ratios were reduced. Deoxynivalenol is shown to alter the intestinal barrier due to impaired gene expression of proteins involved in tight junctions, tissue remodeling and inflammatory reactions [4,40]. Additionally, changes to intestinal structure may occur through villus atrophy and a disruption of the balance between proliferation and apoptosis [41]. From our results, it appears that the majority of intestinal damage due to DON challenge occurs in the jejunum. Avantaggiato et al. [42] utilized an in vitro GI-model for pigs to demonstrate that the majority of DON is absorbed in the jejunum, with lesser amounts in the ileum. Although research has demonstrated that the damages of DON to the gastrointestinal tract morphology of monogastric animals can occur [35,43,44], the results from our current trial show the effects of DON in a pair-feeding setting. As a result, the negative impacts on the morphology of the intestinal tract provide evidence suggesting that damages may be a direct toxic effect of DON rather than a reduction in feed intake.

## 4. Conclusions

This study is one of the first to demonstrate that DON consumption altered L-Trp metabolism through increasing the 5-HT turnover index in the hypothalamus, but no other regions of the brain, when pigs were pair-fed and kept on the same feed intake. As a result of changes in cerebral Trp metabolism, pigs had altered behavioral responses that resulted in increased passive and quiescent behavior. Additionally, the damages to the intestinal tract were shown to be a direct toxic effect of DON rather than a reduction in feed intake. 

## 5. Materials and Methods

### 5.1. Animals and Experimental Diets

The experimental protocol was reviewed and approved by the Institutional Animal Care and Use Committee at North Carolina State University (Raleigh, NC, USA). Pigs were housed individually in solid concrete floor indoor pens (1.42 × 3.86 m) at the North Carolina State University Swine Evaluation Station (Clayton, NC, USA).

Sixteen crossbred barrows (25.6 ± 0.9 kg) at 9 weeks of age were used in this study following a pair-feeding procedure. Pigs were paired based on similar initial body weight into a set of 2 pens (8 pairs) with each pair as a block. Pigs in each pair were randomly allotted to one of 2 dietary treatments (Table 5), representing: a diet without detectible DON (CON) or a diet with 3.8 mg/kg of naturally contaminated DON (MT). Corn naturally contaminated with 18.5 mg/kg of DON and 1.8 mg/kg of 15-acetyl-DON was used in this study to manufacture experimental diets. Mycotoxin analysis was completed by collecting 10 samples from different locations to obtain a representative sample [45]. The 10 samples were combined and thoroughly blended together before 2 subsamples were collected for analysis of mycotoxin content. Mycotoxin contaminants were measured by the North Dakota State Veterinary Diagnostic Laboratory (Fargo, ND, USA) using HPLC. Analysis of complete diets reveled a final concentration of DON at 3.8 mg/kg in the finished feed.

Pigs receiving the MT diet were provided feed ad libitum, and the amount of daily feed consumed by MT pigs dictated the daily feed allotment for their respective control pair. Feed for the pair-fed CON pigs was provided once daily in the morning, receiving the amount consumed by each MT pair-mate on the previous day. Thus, the pigs fed CON began the trial 1 day after the MT pigs and it continued in this way throughout the study so that both treatments were fed for 21 days. This restrictive feeding regimen assured the same amount of feed intake for animals in both treatment groups. Average daily gain, ADFI and G:F ratio were measured during the experimental period. 

### 5.2. Physical Activity

To evaluate the effects of a dietary DON exposure on the behavior of pigs, each pig was continuously recorded for 12 h (from 8:00 a.m. to 8:00 p.m.) in real time on days 0, 7, 14 and 21, according to a previous study by Shen et al. [18]. A previously trained observer watched all recorded videos. Data were analyzed by classifying behavior into lying, sitting, standing, eating and drinking. An instantaneous scan-sampling method with 1 min-interval was used to determine percentage of time spent on various behaviors for every other hour from 8:00 a.m. to 8:00 p.m. [18]. Details of behavioral assessment and definition of each behavior were addressed in Shen et al. [18]. 

### 5.3. Salivary Cortisol 

Saliva samples were collected from each pig for the measurement of salivary cortisol on days 0, 7, 14 and 21. Pigs were allowed to chew on a piece of gauze which became soaked in saliva. Concentrations of salivary cortisol were determined using a commercial RIA kit (Diagnostic Products Corp.) modified for pigs [46]. All samples were tested in duplicate. Intra and inter assay CVs were 4.6 and 4.0%, respectively.

### 5.4. Immunological and Oxidative Stress Parameters

Blood samples were collected from each pig on day 7s and 21 for immunological and oxidative stress parameters. Blood was collected in Monovette tubes (Sarstedt, Newton, NC) containing EDTA to obtain plasma. Samples were centrifuged at 3000 *g* (4 °C) for 15 min, and samples were stored at −80°C until analyzed. 

The immunoglobulins (Ig) IgA, IgG and IgM were measured via an enzyme-linked immunosorbent assay (ELISA, Bethyl, Montgomery, TX, USA) following Chen et al. [47]. Plasma samples were diluted to 1:4000, 1:150,000 and 1:10,000 for IgA, IgG and IgM, respectively. Absorbance was read at 450 nm using a Synergy HT ELISA plate reader (BioTek Instruments, INC, Winooski, VT, USA) and Gen5 data analysis software (BioTek Instruments, INC, Winooski, VT, USA). Samples were quantified relative to the respective standard curve constructed. The ELISA detection limit for IgA was 15.6 to 1000 ng/mL, 7.8 to 500 ng/mL for IgG and 15.6 to 1000 ng/mL for IgM. The cytokine tumor necrosis factor alpha (TNF-α) was also measured by ELISA (R&D Systems, Minneapolis, MN, USA) following [48]. Absorbance was read at 450 nm and 540 using the same equipment as previously described. The detection limit range for TNF-α was 2.8 to 5.0 pg/mL.

Malondialdehyde (MDA) was measured as an indicator of oxidative stress in the form of lipid peroxidation [49,50]. Quantification of MDA was completed using a TBARS assay (Cell Biolabs, INC, San Diego, CA, USA) following Shen et al. [51]. Absorbance was read at 532 nm using the Synergy HT ELISA plate reader, and the MDA content was determined in samples by comparison with the MDA standard curve.

### 5.5. Tissue Collection and Analysis

On day 21, all pigs were euthanized by electrocution to collect samples of the stomach, duodenum, jejunum, ileum, liver, gastric lymph node, lung and brain tissue. Stomach, duodenum, jejunum and ileum samples were fixed in 10% buffered formalin and sent to the North Carolina State University Histopathology Laboratory (College of Veterinary Medicine, Raleigh, NC, USA) for hematoxylin and eosin (H & E) staining and slide preparation. These tissues were analyzed for villus height and crypt depth using an Olympus Vanox microscope (Olympus Corporation, Center Valley, PA, USA) and Spot Advanced software program (SPOT Imaging Solutions, Sterling Heights, MI, USA).

Tissue samples were also placed in liquid nitrogen and were stored at −80°C until further analysis of immunological parameters. Upon analysis, tissues were homogenized in PBS and analyzed for concentrations of IgA, IgG, IgM and TNF-α following protocols as previously described. Tissue samples were diluted at 1:1000 for IgA and IgM and 1:10,000 for IgG. Tissues were not diluted for analysis of TNF-α. Tissue protein concentration of each sample was also analyzed by a bicinchoninic acid (BCA) protein assay (Pierce Biotechnology, Rockford, IL, USA). Liver tissue was diluted at 1:75, lung tissue at 1:50, stomach samples at 1:40, whereas all other tissues were diluted at 1:20. The measured protein concentration was used to determine the amount of immunological subset per gram of protein of each tissue type. 

The brain was quickly removed and the hypothalamus, raphe nuclei and medulla oblongata were dissected and frozen in liquid nitrogen, then stored in −80°C. The frozen brain tissue was weighed and processed to determine concentrations of 5-HT and 5-HIAA, as described by [21]. All samples were tested in duplicate. Intra and inter assay CVs for 5-HT were 4.8% and 6.0%, respectively. Intra and inter assay CVs for 5-HIAA were 5.2% and 5.7%, respectively. Concentrations of 5-HT and 5-HIAA in the hypothalamus were expressed as ng/kg tissue.

### 5.6. Statistical Analysis 

Data for each response were analyzed using the General Linear Model (PROC GLM) of SAS (SAS Inst. Inc., Cary, NC, USA). The design was a randomized complete block design. The individually housed pig was considered as the experimental unit. The pair was the block factor. Differences were considered significant with *p* ≤ 0.05, whereas 0.05 < *p* < 0.10 was considered a tendency.

## Figures and Tables

**Table 1:** Growth performance of pair-fed pigs consuming diets without (CON) or with 3.8 mg/kg deoxynivalenol (MT) from naturally contaminated corn.

	Treatment			
Item	CON	MT	SEM	*p-*Value
**Initial body weight, kg**	25.6	25.5	0.4	0.938
**Average daily gain, g/d**				
day 0 to 7	338	523	38	0.011
day 7 to 14	745	751	32	0.904
day 14 to 21	605	596	26	0.808
day 0 to 21	563	623	14	0.017
**Average daily feed intake** ^1^**, g/d**				
day 0 to 7	1266	1296	19	0.299
day 7 to 14	1614	1646	23	0.351
day 14 to 21	1508	1546	20	0.209
day 0 to 21	1463	1496	17	0.208
**Gain to feed ratio**				
day 0 to 7	0.27	0.40	0.03	0.014
day 7 to 14	0.46	0.46	0.01	0.882
day 14 to 21	0.40	0.39	0.02	0.595
day 0 to 21	0.39	0.42	0.01	0.062

^1^ Pigs were pair-fed between treatments.

**Table 2:** Plasma immune parameters of pair-fed pigs consuming diets without (CON) or with 3.8 mg/kg of deoxynivalenol (MT) from naturally contaminated corn.

	Treatment			
Item	CON	MT	SEM	*p-*Value
**day 7**				
IgA ^1^, µg/mL	2341	2588	138	0.247
IgG ^2^, mg/mL	118	78	16	0.126
IgM ^3^, µg/mL	877	872	42	0.946
TNF-α ^4^, pg/mL	94.7	90.9	4.7	0.581
MDA ^5^, µM	13.1	13.1	1.7	0.991
**day 21**				
IgA, µg/mL	2555	2510	318	0.923
IgG, mg/mL	96	106	23	0.755
IgM, µg/mL	1250	1374	86	0.340
TNF-α, pg/mL	79.7	93.5	11.8	0.436
MDA, µM	12.2	10.7	1.0	0.304

^1^ IgA, immunoglobulin A; ^2^ IgG, immunoglobulin G; ^3^ IgM, immunoglobulin M; ^4^ TNF-α, tumor necrosis factor alpha; ^5^ MDA, malondialdehyde.

**Table 3:** Tissue immune parameters on day 21 of pair-fed pigs consuming diets without (CON) or with 3.8 mg/kg of deoxynivalenol (MT) from naturally contaminated corn.

	Treatment			
Item	CON	MT	SEM	*p-*Value
**IgA ^1^, mg/g protein**				
Stomach	27.51	26.66	3.00	0.846
Duodenum	39.64	43.25	5.14	0.635
Jejunum	20.71	21.84	1.98	0.700
Ileum	34.60	33.37	1.77	0.640
**IgG ^2^, mg/g protein**				
Stomach	53.87	48.42	11.55	0.749
Duodenum	59.13	78.38	12.26	0.304
Jejunum	36.62	37.58	4.92	0.894
Ileum	59.19	69.47	7.23	0.349
**IgM ^3^, mg/g protein**				
Stomach	15.56	14.55	1.45	0.637
Duodenum	9.34	7.92	1.63	0.557
Jejunum	9.11	11.05	1.12	0.260
Ileum	7.40	7.33	0.53	0.926
**TNF-α ^4^, pg/g protein**				
Stomach	709	685	45	0.720
Duodenum	1227	986	93	0.112
Jejunum	716	1038	37	0.004
Ileum	2737	2946	333	0.671
Liver	863	756	113	0.524
Lung	2424	1786	244	0.107
Lymph node	1908	1709	185	0.477

^1^ IgA, immunoglobulin A; ^2^ IgG, immunoglobulin G; ^3^ IgM, immunoglobulin M; ^4^ TNF-α, tumor necrosis factor alpha.

**Table 4:** Tissue morphology on day 21 of pair-fed pigs consuming diets without (CON) or with 3.8 mg/kg of deoxynivalenol (MT) from naturally contaminated corn.

	Treatment			
Item	CON	MT	SEM	*p*-Value
**Stomach**				
Depth of gastric pits, µm	241	175	12	0.006
**Duodenum**				
Villus height, µm	626	576	37	0.365
Crypt depth, µm	550	555	17	0.845
Villus to crypt ratio	1.15	1.04	0.08	0.330
**Jejunum**				
Villus height, µm	553	459	20	0.013
Crypt depth, µm	355	423	17	0.026
Villus to crypt ratio	1.58	1.09	0.05	< 0.001
**Ileum**				
Villus height, µm	525	421	34	0.070
Crypt depth, µm	345	383	19	0.193
Villus to crypt ratio	1.53	1.11	0.06	0.002

**Table 5:** Composition of experimental diets, as-fed basis.

	Treatment ^1^	
Item	CON	MT
**Ingredient, %**		
Ground yellow corn	74.25	59.25
Ground yellow corn with deoxynivalenol ^2^	0.00	15.00
Soybean meal, dehulled	22.00	22.00
L-Lys HCl	0.10	0.10
L-Trp	0.40	0.40
Salt	0.30	0.30
Vitamin premix ^3^	0.03	0.03
Trace mineral premix ^4^	0.15	0.15
Dicalcium phosphate	0.90	0.90
Ground limestone	0.70	0.70
Poultry fat	1.00	1.00
**Calculated composition**		
DM, %	89.6	89.6
ME, Mcal/kg	3.39	3.39
CP, %	17.06	17.06
SID ^5^ Lys, %	0.83	0.83
SID Cys + Met, %	0.51	0.51
SID Trp, %	0.56	0.56
SID Thr, %	0.53	0.53
Ca, %	0.55	0.55
Available P, %	0.23	0.23
Total P, %	0.53	0.53
Deoxynivalenol, mg/kg	0.00	3.00
earalenone, mg/kg	0.00	0.13
**Analyzed composition**		
DM, %	88.7	89.1
CP, %	16.3	16.4
Deoxynivalenol, mg/kg	0.00	3.80

^1^ Pigs fed either a diet without deoxynivalenol (CON) or a diet with 3.8 mg/kg of naturally contaminated deoxynivalenol (MT). ^2^ Corn contained 18.5 mg/kg of DON, 1.8 mg/kg of 15-acetyl deoxynivalenol, 0.8 mg/kg of ZEA, <2 mg/kg of fumonisin and <0.02 mg/kg of aflatoxin. Analysis of mycotoxins in corn was completed by North Dakota State University Veterinary Diagnostic Laboratory (Fargo, ND) by HPLC. ^3^ The vitamin premix provided the following per kilogram of complete diet: 6613.8 IU of vitamin A as vitamin A acetate; 992.0 IU of vitamin D_3_; 19.8 IU of vitamin E; 2.64 mg of vitamin K as menadione sodium bisulfate; 0.03 mg of vitamin B_12_; 4.63 mg of riboflavin; 18.52 mg of D-pantothenic acid as calcium panthonate; 24.96 mg of niacin; 0.07 mg of biotin. ^4^ The trace mineral premix provided the following per kilogram of complete diet: 4.0 mg of Mn as manganous oxide; 165 mg of Fe as ferrous sulfate; 165 mg of Zn as zinc sulfate; 16.5 mg of Cu as copper sulfate; 0.30 mg of I as ethylenediamine dihydroiodide; and 0.30 mg of Se as sodium selenite. ^5^ Standardized ileal digestible.

## Data Availability

Not applicable.
